# Oxidant/Antioxidant Status Is Impaired in Sepsis and Is Related to Anti-Apoptotic, Inflammatory, and Innate Immunity Alterations

**DOI:** 10.3390/antiox11020231

**Published:** 2022-01-25

**Authors:** Marianna Miliaraki, Panagiotis Briassoulis, Stavroula Ilia, Kalliopi Michalakakou, Theodoros Karakonstantakis, Aikaterini Polonifi, Kalliopi Bastaki, Efrossini Briassouli, Konstantinos Vardas, Aikaterini Pistiki, Maria Theodorakopoulou, Theonymfi Tavladaki, Anna-Maria Spanaki, Eumorfia Kondili, Helen Dimitriou, Maria Venihaki, Sotirios Tsiodras, Dimitrios Georgopoulos, Marina Mantzourani, Serafeim Nanas, Apostolos Armaganidis, George L. Daikos, Ioannis Papassotiriou, George Briassoulis

**Affiliations:** 1 Pediatric Intensive Care Unit, University Hospital of Heraklion and School of Medicine, University of Crete, 71110 Heraklion, Greece; marianmyl@yahoo.gr (M.M.); briaspan@hotmail.com (P.B.); stavroula.ilia@uoc.gr (S.I.); med10p1130084@med.uoc.gr (K.B.); efi.tavladaki@gmail.com (T.T.); spanakam@yahoo.gr (A.-M.S.); 2 Department of Clinical Biochemistry, “Aghia Sophia” Children’s Hospital, 11527 Athens, Greece; kleiwmamy@gmail.com (K.M.); docb@paidon-agiasofia.gr (T.K.); ipapassotiriou@gmail.com (I.P.); 3 First Department of Internal Medicine-Propaedeutic, National and Kapodistrian University of Athens, 11527 Athens, Greece; kpolo@med.uoa.gr (A.P.); efroelesar@hotmail.com (E.B.); mantzourani@gmail.com (M.M.); gdaikos@med.uoa.gr (G.L.D.); 4 First Critical Care Department, Evangelismos University Hospital, National and Kapodistrian University of Athens, 10676 Athens, Greece; costas_vardas@yahoo.gr (K.V.); sernanas@gmail.com (S.N.); 5 4th Department of Internal Medicine, Attikon University Hospital, National and Kapodistrian University of Athens, 12462 Athens, Greece; aipistiki@gmail.com (A.P.); tsiodras@med.uoa.gr (S.T.); 6 2nd Department of Critical Care, Attikon University Hospital, Medical School, National and Kapodistrian University of Athens, 12462 Athens, Greece; mariatheodor10@gmail.com (M.T.); aarmag@med.uoa.gr (A.A.); 7 Department of Intensive Care Medicine, University Hospital of Heraklion and Medical School, University of Crete, 71110 Heraklion, Greece; kondylie@uoc.gr (E.K.); georgop@med.uoc.gr (D.G.); 8 Laboratory of Child Health, School of Medicine, University of Crete, 71003 Heraklion, Greece; lena.dimitriou@uoc.gr; 9 Department of Clinical Chemistry, School of Medicine, University of Crete, 71003 Heraklion, Greece; venihaki@med.uoc.gr

**Keywords:** oxidative stress, antioxidant status, apoptosis, interleukins, heat shock proteins, TOS, TAC, innate immunity

## Abstract

Oxidative stress is considered pivotal in the pathophysiology of sepsis. Oxidants modulate heat shock proteins (Hsp), interleukins (IL), and cell death pathways, including apoptosis. This multicenter prospective observational study was designed to ascertain whether an oxidant/antioxidant imbalance is an independent sepsis discriminator and mortality predictor in intensive care unit (ICU) patients with sepsis (*n* = 145), compared to non-infectious critically ill patients (*n* = 112) and healthy individuals (*n* = 89). Serum total oxidative status (TOS) and total antioxidant capacity (TAC) were measured by photometric testing. IL-6, -8, -10, -27, Hsp72/90 (ELISA), and selected antioxidant biomolecules (Ζn, glutathione) were correlated with apoptotic mediators (caspase-3, capsase-9) and the central anti-apoptotic survivin protein (ELISA, real-time PCR). A wide scattering of TOS, TAC, and TOS/TAC in all three groups was demonstrated. Septic patients had an elevated TOS/TAC, compared to non-infectious critically ill patients and healthy individuals (*p* = 0.001). TOS/TAC was associated with severity scores, procalcitonin, IL-6, -10, -27, IFN-γ, Hsp72, Hsp90, survivin protein, and survivin isoforms -2B, -ΔΕx3, -WT (*p* < 0.001). In a propensity probability (age-sex-adjusted) logistic regression model, only sepsis was independently associated with TOS/TAC (Exp(B) 25.4, *p* < 0.001). The AUC_TOS/TAC_ (0.96 (95% CI = 0.93–0.99)) was higher than AUC_TAC_ (z = 20, *p* < 0.001) or AUC_TOS_ (z = 3.1, *p* = 0.002) in distinguishing sepsis. TOS/TAC, TOS, survivin isoforms -WT and -2B, Hsp90, IL-6, survivin protein, and repressed TAC were strong predictors of mortality (*p* < 0.01). Oxidant/antioxidant status is impaired in septic compared to critically ill patients with trauma or surgery and is related to anti-apoptotic, inflammatory, and innate immunity alterations. The unpredicted TOS/TAC imbalance might be related to undefined phenotypes in patients and healthy individuals.

## 1. Introduction

Oxidative stress is common in critical illness, as a result of the generation of oxygen and nitrogen-derived free radicals and disruption of redox signaling or antioxidant control systems, including the decline in key antioxidant compounds [1]. According to the “redox hypothesis”, the oxidation of intra- or extracellular thiols, along with reactive oxygen and nitrogen-oxygen species, leads to mitochondrial dysfunction, tissue injury, organ failure, or death [2]. Experimental studies indicate that T helper (Th)1 and Th2 cell responses are controlled by intracellular glutathione redox status in dendritic cells, through cytokine-mediated upregulation [3]. Oxidative stress is defined as an imbalance between the production of reactive oxygen species (ROS) and the antioxidant capacity of an organism. Total oxidative status (TOS) represents the oxidizing state of patients and is usually estimated through direct measurement of relatively stable ROS family members, such as hydrogen peroxide (H_2_O_2_), peroxyl radicals, and DNA damage markers. TOS may be estimated indirectly by examining the oxidative damage these radicals cause to lipids, proteins, or nucleic acids. An imbalance in cellular redox homeostasis is regulated by enzymatic or non-enzymatic antioxidant machinery systems, expressed by the total antioxidant capacity (TAC) [4]. Current research focuses on the evaluation of possible correlations between TOS or TAC, as well as clinical severity, in intensive care unit (ICU) patients with sepsis [5,6].

Sepsis is a complex clinical condition defined as the detrimental immunological host response to infection, interwoven with excessive inflammatory and oxidative stress cascades, which counteract and ultimately serve as cell death signals. Apoptotic cell death in sepsis mainly concerns lymphocytes, intestinal and lung epithelial cells, and predisposition to immune system weakening and higher mortality risk [7]. This programmed cell death is driven through distinct apoptotic processes, such as pyroptosis, autophagy, or ferroptosis [8]. Intrinsic apoptosis is a mitochondrion-centered cell death that is mediated by mitochondrial outer membrane permeabilization, resulting in apoptosome formation, activation of caspase-9, and subsequent activation of effector caspase-3 [9].

The septic start point is characterized by initial pathophysiological alterations collectively known as an uncontrolled inflammatory storm, leading to overproduction of oxidants [10,11]. It has already been shown that the integrity of the immune and antioxidant potential is of critical importance for the resolution of sepsis [6,12]. There is also a large body of evidence demonstrating that heat shock proteins (Hsp), which represent chaperons of the innate immune system, are readily induced for protection against oxidative stress in a wide variety of stress conditions, including sepsis [13]. It has been previously shown that serum Hsp72 levels are modulated according to the patient oxidant status, and increased serum Hsp72 levels are associated with mortality in sepsis [14].

Crosstalk between early-onset immune and inflammatory response, oxidative stress, and apoptosis might be related to sepsis-induced multi-organ disorders. However, the interplay between redox imbalance and (1) apoptotic-antiapoptotic pathways, (2) innate immunity mediators, such as chaperone Hsp, and (3) inflammatory biomolecules, such as interleukins, in ICU patients, and its effect on their clinical course and outcome has not been delineated yet.

This multicenter prospective observational study was designed to test the hypothesis that an oxidant/antioxidant imbalance is an independent sepsis discriminator and mortality predictor in critically ill patients with sepsis, compared to other intensive care patients in acute stress. A secondary aim was to examine the association of early-onset oxidative burst with the severity of illness, inflammatory, innate immunity, and apoptotic/anti-apoptotic response in septic patients, compared to non-infectious critically ill and healthy individuals.

## 2. Materials and Methods

### 2.1. Patients

Adult patients (>18 years old) consecutively admitted with early-onset (<48 h) sepsis/septic shock (S) or non-infectious critical illness (I) were eligible for enrollment. The sepsis group included patients with an identified source of infection and Sequential Organ Failure Assessment (SOFA) score > 2 [15], according to the updated Sepsis-3 definition for adults, while the septic shock criteria (need for vasopressor support to maintain MAP > 65 mmHg and lactate levels > 2 mmol/L) were used to identify patients with septic shock [16]. The non-infectious critical illness group included trauma or post-surgical patients who met at least two of the four conventional criteria for systematic inflammatory response syndrome (SIRS) [17] and represented the first control group (ICU control) (I). Healthy volunteers (H) represented the second control group (healthy individuals). Exclusion criteria were malignancy, immune deficiency, and late-sepsis or SIRS > 24 h after admission. The per group contribution of each participating center was equal.

### 2.2. Primary and Secondary Outcomes

Demographic data, comorbidities, clinical signs, and outcome hardpoints were recorded through patients’ electronic medical databases. The primary outcomes were mortality and severity of illness, assessed through standardized severity scoring systems on admission. The Acute Physiology and Chronic Evaluation-II (APACHE II), Multiple Organ Dysfunction (MODS), Simplified Acute Physiology Score-III (SAPS III), and SOFA score [15] were recorded on admission. ICU length of stay (LOS) and multiple inflammatory indices were assessed as secondary outcomes. Procalcitonin (PCT), lactate, CRP, and various organ function parameters were also recorded.

### 2.3. Assays

Regarding oxidative stress assays, arterial blood samples from indwelling catheters were obtained within the first hours of ICU admission, and they were immediately centrifuged at 3000 rpm for 10 min. Serum was then stored at −20 °C until analysis, according to the manufacturer’s instructions. Total lipid peroxides quantification was used to assess TOS, based on the reaction of peroxidase with peroxides in the sample, using tetramethylbenzidine as a chromogen substrate measured at 450nm wavelength (PerOx kit, Immundiagnostik AG, Stubenwald-Allee 8a, and D 64,625 Bensheim, Germany). The intra-assay and inter-assay CVs were 3.1% and 5.1%, respectively; the detection limit was 7 mmol/L. TAC was measured by a colorimetric test system (ImAnOx kit, Immundiagnostik AG, Stubenwald-Allee 8a, and D 64,625 Bensheim, Germany) [18,19], which reflects the sum of all antioxidant components, through an enzymatic reaction of sample antioxidants with a defined amount of exogenously provided H_2_O_2_. The antioxidants in the sample eliminate a certain amount of the provided H_2_O_2_, which involves the conversion of tetramethylbenzidine to a colored product measured at 450nm wavelength. The intra-assay CV was 1.6%, the inter-assay CV was 2.0%, and the detection limit was 130mmol/L. Because active ROS are metabolized rapidly in vivo, their evaluation represents an extremely difficult task. Even though there is no harmonization of various techniques, several indirect methods are used for TOS and TAC assessment, mainly for research purposes [20,21]. Previous studies have measured non-protein TAC (uric acid, bilirubin, vitamin C, polyphenols) but not proteins constituting the main serum antioxidant component [22], or only selected TAC components (2,2′-azino-di-(3-ethylbenzthiazoline sulphonate) (ABTS)) [23]. The contribution of serum uric acid in the total radical-trapping antioxidant parameter (TRAP) is higher than 50%, significantly influencing TAC levels [24]. Similarly, οxidative damage can be estimated by total oxidative status or by quantifying the oxidative by-products of lipids and proteins [24]. Τhe thiobarbituric acid-reactive substance method (TBARS) assay used to measure TOS does not specifically measure malondialdehyde or lipid peroxidation, while the presence of other aldehydes may confound the results [25]. In addition, lipid peroxidation is not specific to sepsis and may be affected by comorbid conditions, diet, and lifestyle behaviors, reporting disappointing predictive utility [20]. Studies using these methods had neither included disease and/or healthy controls nor did they simultaneously measure TOS, TAC, and TOS/TAC, reporting weak to moderate sensitivities/specificities in predicting mortality. Measurement of the overall effect of oxidants (TOS) or antioxidants (TAC) seems to be more accurate due to complex interactions between these biomolecules [4]. Meaningful results from this methodology have repeatedly been published in previous studies [18,26,27,28]. Whole blood was used for determining red cell oxidized/reduced glutathione (GSSG and GSH, respectively) by reverse-phase HPLC with fluorimetric detection (excitation at 385 nm and emission at 515 nm). This method measures the total GSH and reduced GSH. HPLC procedures were performed through the HP 1100 Series HPLC system (Hewlett Packard, Agilent Technologies, INC. 5301 Stevens Creek Blvd, Santa Clara, CA 95051, USA). The determination of Zn in serum was performed by atomic absorption spectrometry (Perkin Elmer Analyst 800 Atomic Absorption Spectrometer, 940 Winter St, Waltham, MA 02451, USA).

Survivin protein (Elabscience Biotechnology, Inc., E-EL-H1584, Houston, TX 77079, USA), along with caspase-3 (Elabscience Biotechnology, Inc., E-ELH0017, Houston, TX 77079, USA) and caspase-9 (Elabscience Biotechnology, Inc., E-EL-H0663, Houston, TX 77079, USA), was also measured in serum through ELISA quantification analysis, according to manufacturers’ instructions. The E-EL-H1584 detects survivin-wild-type protein (isoform 1, Baculoviral IAP repeat-containing protein 5, length 142, also known as alpha, apoptosis inhibitor 4, or apoptosis inhibitor survivin) (UniParc, identifier: O15392-1), chosen as the canonical sequence (Elabscience Biotechnology, Houston, TX 77079, USA). Accordingly, survivin protein in this study only represents isoform 1 (WT). The E-EL-H0017 and E-EL-H0663 kits detect pro-caspase-3 and -9, their cleaved subunits, and their active heterodimer formed by p17 and p12 subunits, and they are suitable for testing cleaved caspase-3 and -9, respectively. All specimens were assayed twice. The detection range was around 1.58 to 9.96 pg/mL for survivin, 0.31–20 ng/mL for caspase-3, and 1.563–100 ng/mL for caspase-9.

The transcriptional expression of survivin was quantified through real-time quantitative polymerase chain reaction for the different survivin splice variants (wild type-WT, Δex3, 2B, 3B) in peripheral blood leukocytes. Blood samples were obtained within 24 h of admission to the ICU and then stored at −80 °C until total RNA extraction. The intracellular cumulative transcriptional mRNA expression of survivin in white blood cells was assessed through real-time quantitative polymerase chain reaction (qPCR), which was performed on the complementary DNA (cDNA), using reverse primers and hybridization probes, specific for the genes of interest for each splice variant of survivin (RNA extraction and reverse transcription for cDNA synthesis). New forward and reverse primers for each variant were designed to prevent non-specific amplifications [29]. Total mRNA was isolated from blood samples and K652 human cell line, using the Trizol reagent (Monarch Total RNA Miniprep Kit, NEB #T2010, New England Biolabs, Herts SG4 0TY, UK) and reverse transcription was performed using an iScript cDNA synthesis kit (BioRad, 1708891, Hercules, CA 94547, USA) according to the manufacturer’s instructions. qRT-PCR analysis was carried out using iTaq SYBR Green Universal Supermix (BioRad, 1725122) in a Thermal Cycler real time PCR detection system (BioRad CFX96, Hercules, CA 94547, USA). Melting curve experiments had previously established that the fluorescence signal for each amplicon was derived from the products only, and no primer dimers were found. Absolute quantification was applied by calculating the ratio of the number of the molecules (copies/μL) of the target gene to the number of the molecules (copies/μL) of the reference gene (β-actin). The internal standards were not based on the cDNA library but consisted of tenfold serial dilutions of K562 cell line cDNA. The real-time PCR efficiencies were calculated from the slope. Amplification efficiency was similar (1684–1986) between the target and the reference gene, respectively. The evaluation for the proper size and purity of PCR samples was performed through electrophoresis on agarose gels. These data were related to severity of illness scoring systems and outcome (APACHE and SOFA score).

Extracellular Hsp72 and Hsp90 expression were quantified by ELISA commercial kits (Enzo Life Sciences, Lausanne, Switzerland). The sensitivities of the assays were 50 ng/mL for Hsp90 and 90 ng/mL for Hsp72. Interleukins (IL)-6, -8, -10, -27, and IFN-γ in serum were also evaluated by available ELISA kits according to manufacturer’s instructions (Invitrogen, ThermoFisher Scientific, Carlsbad, CA 92008, USA). The sensitivities of the assays were <2 pg/mL for IL-6, <2 pg/mL for IL-8, and <1 pg/mL for IL-10. Extracellular Hsp72 and Hsp90 expression were also quantified by ELISA commercial kits (Enzo Life Sciences, Lausanne, 4415 Switzerland).

Blood samples for plasma glutamine determination were obtained in heparinized tubes, kept on ice, and centrifuged within 30 min (2000 g for 10 min at 4 °C). The analyses were performed by automated online high-performance liquid chromatography (HPLC) after deproteinization with trichloroacetic acid (TCA) 30%. The amino acid was separated in an amino-acid analyzer (Hitachi Aminoacid analyzer L 8900, 24-14 Nishi-Shimbashi 1-chome, Minato-ku, Tokyo 105-8717, JP) at a constant flow on a high-resolution cation-exchange column (Neolab S.A, Athens 11527, Greece). The post-column derivatization reaction with the ninhydrin reagent was carried out at 135 °C, and the absorbance of the reaction products was read at both 570 and 440 nm.

As far as routine laboratory measurements are concerned, CRP assays were performed on Beckman Coulter AU Analyzers (Beckman Coulter, 2470 Faraday Ave, Carlsbad, CA 92010, USA). CRP levels > 0.8 mg/dL were considered abnormal. PCT was measured by a latex enhanced immunoturbidometric assay (Diazyme-PCT Assay, Diazyme Laboratories, Inc. 12889 Gregg Ct. Poway, CA 92064, USA). Detection threshold was 0.1 ng/mL; PCT levels > 0.5 ng/mL were considered abnormal (laboratory cut off values).

### 2.4. Statistical Analyses

To calculate an adequate sample size, we used the G*Power statistical power calculator (Heinrich Heine Universität, Dusseldorf, Germany). Statistical test ANOVA (F-test): Fixed effects special, main effects, and interactions; power = 0.80, alpha = 0.05, effect size medium (f = 0.25). The calculated total sample size was 200 (all groups), critical F = 2.42, non-centrality parameter λ = 12.5, denominator df = 195. Levene’s test of the homogeneity of group variances was used to determine the data distribution from measured variables.

Descriptive statistics of serum markers are presented for the three study groups (S, I, and H groups). Categorical variables are described in absolute values and frequency. Quantitative variables are expressed in mean and standard deviation (normal distribution), or in median and interquartile range (non-normal distribution). The Shapiro–Wilk test of the homogeneity of group variances was used to determine the data distribution from measured variables. ANOVA with Tukey post hoc tests, or the Kruskal–Wallis independent samples test with multiple comparison analyses, using post hoc Dunn’s pairwise tests with Bonferroni corrections, and the Mann–Whitney U-tests were used to perform comparisons among parametric or nonparametric groups, as appropriate. Between-group comparisons were conducted using the χ^2^ test for categorical parameters and Spearman’s correlation coefficient for correlation between two continuous variables. A linear regression model (backward method) was adopted to examine whether any of the studied variables are independently associated with TOS or TAC. We first used univariate models to test all clinical and laboratory variables related to TOS/TAC, with just one explanatory variable at a time; afterwards, all variables that had shown a relaxed *p* value of less than or equal to 0.1 were included in the multivariate models. To evaluate sepsis discriminators, the areas under the receiver operating characteristic curves (AUROCs) for variables significantly differing between septic and non-septic ICU patients were calculated. The “optimal” cut off point of TOS/TAC for the best sensitivity-specificity combination was calculated by the Youden index (J) and confirmed by the Closest to (0,1) Criteria (ER) [30]. Pairwise comparisons of every 2 ROC curves were performed using the z test. Based on the “cut off” line, the scattering of individual TOS/TAC calculated cases was then estimated. A propensity probability binary logistic regression model, adjusted for predicted probabilities of the covariates age and sex, was adopted to examine whether any of the studied variables were independently associated with the TOS/TAC ratio. The “optimal” cut off point defined the dependent binary variable TOS/TAC. Independent variables included the propensity score of covariates age and sex (predicted probability), diagnostic categories (sepsis, non-septic ICU patients, and healthy controls), the severity of illness (SOFA score), and the early onset alarmin Hsp72. To evaluate outcome predictor values, AUROCs for variables significantly differing between survivors and non-survivors were calculated. Statistical analysis software (version 26, SPSS, Chicago, IL, USA) was used for all analyses.

## 3. Results

### 3.1. Patient Demographic Characteristics

This study included 145 patients with sepsis/septic shock (S) (85 males (59%) and 60 females (41%), 112 patients with non-infectious critical illness (I) (80% males), and 89 healthy controls (H) (56% males). The demographic and clinical characteristics of studied patients are shown in Table 1. Common comorbidities (obesity, diabetes, allergy, chronic illnesses) did not differ among groups. Markers of systemic inflammation (CRP and procalcitonin) and stress (glucose) were significantly elevated in sepsis.

### 3.2. Group Differences

**Figure 1:**
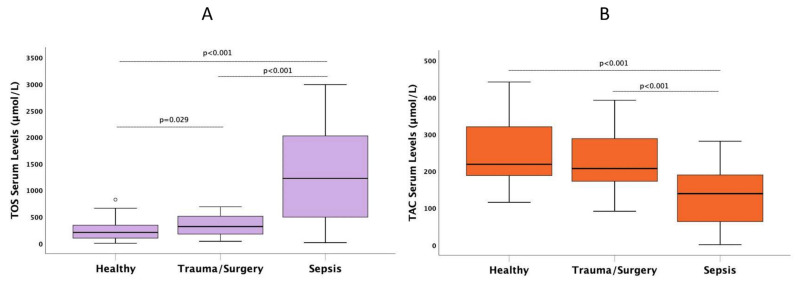
Serum median levels of (**A**) total oxidative stress (TOS) and (**B**) total antioxidant capacity (TAC) in septic patients in comparison to patients with non-infectious (trauma/surgery) and healthy controls. The bold black line in box plots indicates the median per group, the bottom of the box indicates the 25th percentile, and the top of the box represents the 75th percentile; the T-bars (whiskers) and horizontal lines show minimum and maximum values of the calculated non-outlier values. Connectors indicate significantly higher levels in sepsis (post hoc Dunn’s pairwise tests with Bonferroni corrections).

Group differences of studied biomolecules are presented in Table 2. TOS/TAC was increased in S compared to I and H (*p* < 0.001). TOS was elevated in septic patients (S) compared to control groups I and H (median (IQR) values of 1222 (493–2022) versus 315 (175–510) versus 206 (99–340) μmol/L, *p* < 0.001) (Figure 1A). TAC was decreased in S compared to I and H (median (IQR) values of 138 (64–190) versus 207 (172–288) versus 218 (188–320) μmol/L, *p* < 0.001) (Figure 1B).

**Figure 2:**
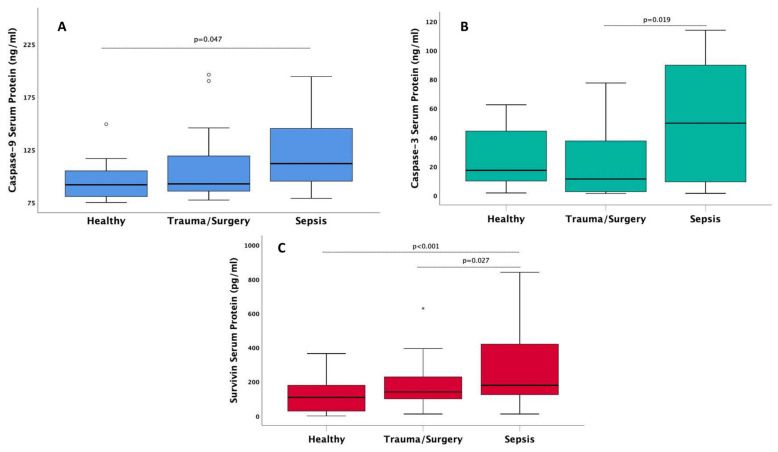
Boxplots of serum median levels of (**A**) apoptotic caspase-9 (intrinsic pathway inducible caspase), (**B**) caspase-3 (effector caspase), (**C**) survivin (antiapoptotic protein) in septic patients in comparison to patients with non-infectious critical illness (trauma/surgery) and healthy controls. The bold black line in box plots indicates the median per group, the bottom of the box indicates the 25th percentile and the top of the box represents the 75th percentile; the T-bars (whiskers) and horizontal lines show minimum and maximum values of the calculated non-outlier values. Solid circles represent outliers, stars extremes. Connectors indicate significantly higher levels in sepsis (post hoc Dunn’s pairwise tests with Bonferroni corrections).

Early-onset serum levels of the induced by the intrinsic mitochondrial pathway caspase-9 (*p* = 0.014), the effector of intrinsic and extrinsic pathways caspase-3 (*p* = 0.021), and anti-apoptotic protein survivin (*p* < 0.001) were found elevated in S compared to I or H (Table 2). Between group post-hoc differences of caspase-9, caspase-3, and survivin are shown in Figure 2.

Three survivin splice variants were increasingly expressed in peripheral blood leukocytes in S, compared to I and H (Survivin-WT, -2B, ΔEx3; *p* < 0.001). Survivin-3B was higher in I compared to S and H (*p* = 0.029) (Table 2).

Inflammatory biomarker and extracellular Hsp levels are presented in Table 2. TNF-α (*p* = 0.016), IL-6 (*p* < 0.001), IL-10 (*p* < 0.001), IFN-γ (*p* = 0.007), and IL-27 (*p* = 0.009) were increased in S compared to control groups I and H. IL-17 and IL-8 did not differ between groups. Hsp72 and Hsp90 were increased in S compared to control groups I and H (*p* < 0.001).

### 3.3. TOS/TAC Ratio Correlations

Neither TOS/TAC ratio nor TOS or TAC was correlated to age in sepsis (Table 3). Bivariate correlations revealed positive correlations of the TOS/TAC ratio with clinical severity scores, inflammation markers (procalcitonin, IL-6, -10, -27, IFN-γ), Hsp72 and Hsp90, the anti-apoptotic survivin protein, and survivin isoforms -2B, -ΔΕx3, -WT. Caspase-9 was positively related with glutathione (*r_s_* = 0.40, *p* = 0.022). TOS was negatively related with Zn (*r_s_* = −0.37, *p* = 0.001), but no correlation was found regarding caspases or survivin-3B (Table 3). Markers of systemic inflammation (CRP and procalcitonin) were positively related to TOS, and negatively to TAC.

### 3.4. Oxidant—Antioxidant Independent Associations

A linear regression analysis model (backward method) showed that Hsp72 (Beta = 0.32, *p* = 0.001) and IL-6 (Beta = 0.34, *p* = 0.008) were independently associated with TOS, whereas survivin protein (Beta = −0.22, *p* = 0.017) and Hsp72 (Beta = −0.24, *p* = 0.01) were inversely associated with TAC.

**Figure 3:**
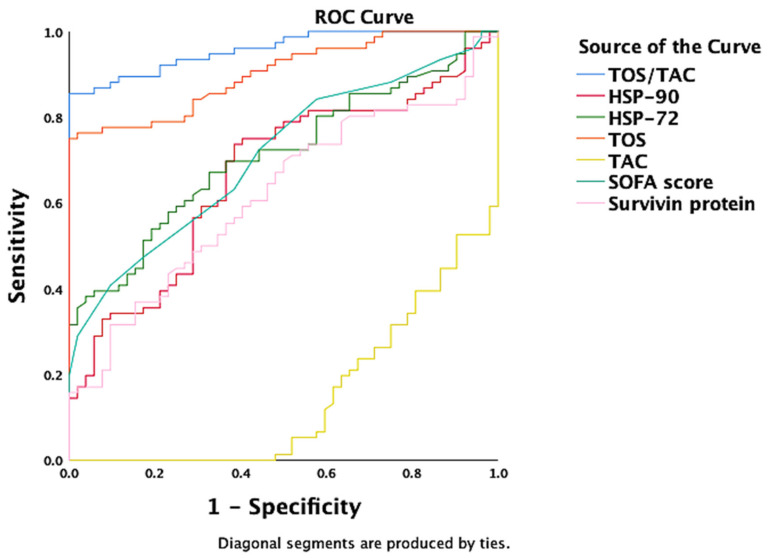
ROC curve for discriminating sepsis among critically ill patients. Independently, TOS/TAC, TOS, Hsp72 and Hsp90, SOFA score, survivin protein, and repressed TAC were independent discriminators of sepsis (AUROC > 60%, *p* < 0.05). The TOS/TAC ratio achieved the best AUROC (0.96 (95% CI = 0.93–0.99), *p* < 0.001).

In an ROC analysis, TOS/TAC, TOS, Hsp72 and Hsp90, SOFA score, survivin protein, and repressed TAC were independent discriminators of sepsis (Figure 3). The TOS/TAC ratio achieved the best AUROC (0.96 (95% CI = 0.93–0.99), *p* < 0.001) (Table 4). Receiver operating characteristic curve analysis revealed that the area under the AUC_TOS/TAC_ was significantly higher than AUC_TAC_ (z = 20, *p* < 0.001) or AUC_TOS_ (z = 3.1, *p* = 0.002) in distinguishing sepsis from control groups (including non-septic critical illness control and the healthy control). The optimal TOS/TAC cut off point value calculated by the Youden index (J) and confirmed by the Closest to (0,1) Criteria (ER) was 3.963 (sensitivity 85%, specificity 98%).

In a propensity probability (age-sex-adjusted) logistic regression model, only sepsis was independently associated with TOS/TAC (binary variable low < 3.963 > high, defined by the “optimal” cut off point of TOS/TAC value), demonstrating an odds ratio of 25 (Exp(B) 25.4, *p* < 0.001). Neither the propensity score (predicted probability of covariates age and sex, *p* = 0.385) nor SOFA score (*p* = 0.592) or Hsp72 (*p* = 0.250), shown to be an independent variable in univariate analysis, was independently associated with TOS/TAC changes (Supplementary Table S1).

**Figure 4:**
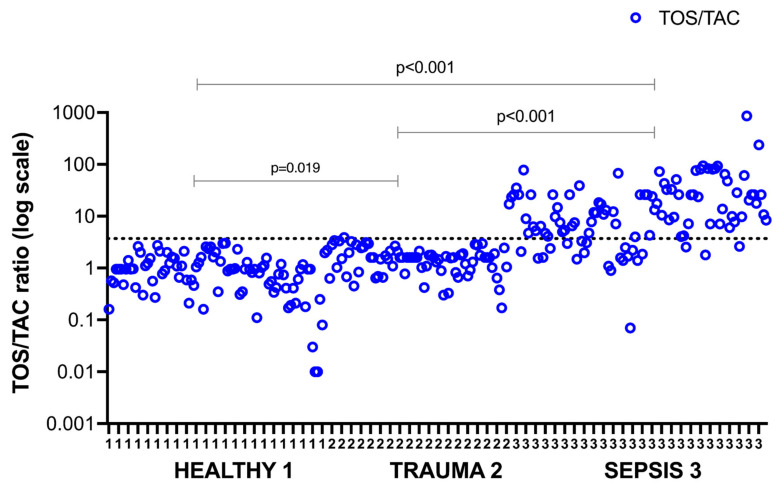
Recordings of individual TOS/TAC ratios (open blue dots) in the sepsis and the two control groups (logarithmic scale). The TOS/TAC cut off point is depicted by the dotted line. Horizontal lines indicate post hoc differences between groups.

TOS/TAC ratios of individual cases above and below the cut off value (dotted line) are shown in Figure 4. A wide scattering of oxidant-antioxidant balance points in all three groups is demonstrated, indicating non-linearly dependent TOS/TAC changes. The TOS/TAC imbalance is distributed above the line in sepsis (higher ratios), and below the line in the non-septic, ICU control group and healthy individuals (*p* < 0.001).

### 3.5. Predictors of Mortality

**Figure 5:**
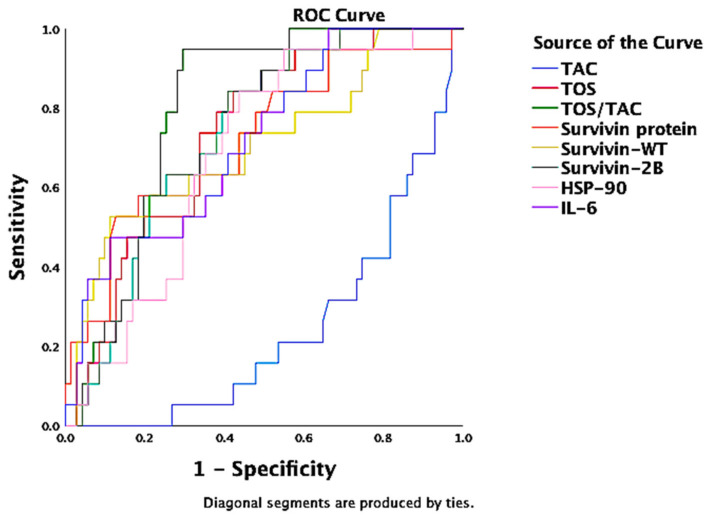
ROC curve for predicting mortality in septic patients. Independently, TAC (inverse prediction), TOS, survivin protein, IL-6, survivin transcript variants -2B and -WT (positive prediction) achieved significant receiver operating characteristic curves—AUROC of >0.70 (*p* < 0.004). The TOS/TAC ratio achieved the best AUROC (0.80, *p* < 0.001).

TOS, IL-6, IL-27, Hsp72, and survivin protein serum concentrations were elevated in the mortality group of septic patients, compared to survivors (Table 5). TOS/TAC, TOS, survivin transcript variants -WT and -2Β, Hsp90, IL-6, survivin protein, and repressed TAC were strong (AUROC > 0.70) independent predictors of mortality (Figure 5). The TOS/TAC ratio achieved the best AUROC (0.80 (95% CI = 0.71–0.89), *p* < 0.001) (Table 6).

## 4. Discussion

Excessive levels of oxidative stress, along with a decreased antioxidant capacity, seem to characterize the redox imbalance in sepsis, disrupting cellular homeostasis and leading to cell death. In the present study, we showed that sepsis is independently associated with an increased TOS/TAC imbalance compared to non-septic ICU patients. In addition, we demonstrated that the oxidant-antioxidant imbalance is an early-onset sepsis discriminator associated with mortality and accompanied by augmentation of the intrinsic mitochondrial apoptotic pathway caspase-9 and the effector of intrinsic and extrinsic apoptotic pathways caspase-3. Finally, we showed that TOS/TAC changes are affected by various inflammatory biomolecules, the innate immune system alarmins Hsp72 and Hsp90, the anti-apoptotic protein survivin, and sepsis-related severity. Multiple reviews on the topic conclude that the mitochondrial dysfunction in sepsis is related to oxidative stress and impairment of oxygen utilization by cells, ultimately resulting in cellular energy failure [31]. Moreover, the favorable results of antioxidant therapies are being highlighted in several clinical trials with septic patients [3,32].

*Oxidative stress:* Oxidative stress represents a common final pathway related to inflammation, ischemia-reperfusion, multiple organ failure, and sepsis [33]. This is the first report showing that the acute stress-related increased expression of Hsp alarmins, cytokines, and survivin in sepsis are independently associated with TOS induction, leading to TOS/TAC imbalance, which is a strong sepsis discriminator and predictor of mortality. These results confirm findings of an animal model, showing an early-onset multiorgan profile of oxidative damage when comparing lethal to non-lethal sepsis [34]. Similar to our results, Hsiao et al. recently demonstrated that oxidative stress, along with a weakened antioxidant defense, reflects the development of sepsis and that serial measurement of TOS and TAC biomarkers could be promising prognostic predictors during the septic course [35]. Our study is also in consonance with other recent reports of an altered equilibrium among oxidants and antioxidants that seems to determine the severity of critical illness, both in sepsis and in traumatic injuries [6,36,37]. In contrast, Lorente has shown increased oxidant stress by measuring biomarkers of oxidative stress-induced lipid peroxidation in sepsis [25]. He also reported increased TAC in septic and trauma patients [23,38,39,40], with no discrimination on the oxidant-antioxidant balance between sepsis and other critically ill ICU patients. A previous report also found that the independent testing of TOS or TAC may not accurately reflect the patient’s oxidative stress. The reason is a dynamic balance between TOS and TAC. The authors conclude that TOS and TAC should be determined simultaneously for an overall evaluation of the oxidative status of a subject [22].

*Total antioxidant capacity:* Total antioxidant capacity (TAC) and erythrocyte total reduced glutathione (GSH) concentration are significantly decreased in sepsis, suggesting that sepsis causes an imbalance between cellular antioxidant and oxidative states [41]. In our double-comparative study, we showed, for the first time, that TAC is decreased in sepsis compared to non-infectious critically ill patients and to healthy individuals. We further showed that repressed TAC levels, along with induced TOS, innate immunity, and inflammatory and anti-apoptotic activity, are independently associated with mortality in sepsis. Similar to our results, recent studies have demonstrated repressed levels of antioxidant enzymes in critically ill septic patients [6,35,36]. In contrast, previous studies reported elevated TAC levels in septic patients associated with mortality [23,24,38]. Regarding TAC in traumatic critically ill patients, recently published results are contradictory. Lorente et al. found a significant association between elevated serum TAC and risk of mortality in severe traumatic brain injury patients [39,40], while other researchers reported significantly decreased TAC in trauma patients [42,43]. Comparing two ICU groups, we found repressed TAC levels in sepsis but not in trauma patients. Integrating the results of various studies remains difficult, which could probably be attributed to different methodologies in measuring antioxidant status. These discrepancies also highlight the importance of establishing well-standardized techniques for measuring oxidative stress and antioxidant defenses in well-designed controlled studies.

Regarding other protective systems, the levels of multiple antioxidant micronutrients, such as vitamins A, C, and D, selenium, α-tocopherol, lycopene, or ascorbic acid, have been reported to be decreased in sepsis [44,45,46]. In our study, Zn was also decreased in ICU patients and was negatively related to TAC. However, glutathione was increased in the septic group and glutamine did not differ among groups. Although multiple promising studies have suggested a variety of strategies for repletion of antioxidants to prevent substantial energy deficits or apoptosis and ameliorate the outcome of septic patients, recent reports have questioned the epiphenomenon of decreased individual antioxidants in sepsis [47,48,49].

*TOS/TAC imbalance*: This study is the first in ICU patients to show that the TOS/TAC ratio is a better sepsis discriminator than TOS or TAC, demonstrating an odds ratio of 25. By including a non-septic ICU control group, we followed the research design of cancer studies showing that TOS/TAC is significantly higher than TAC alone in distinguishing studied patients from control groups (including disease and healthy controls) [50]. In addition, the results of our study showed that the TOS/TAC balance predicted mortality better than TOS or TAC alone, survivin protein, survivin transcript variants, Hsp90, or IL-6. Cell oxidation-antioxidant system may remain in dynamic equilibrium when TOS and TAC simultaneously increase or decrease, so these values alone will not provide the individual’s oxidative stress status. Therefore, it has been suggested that TOS and TAC should be determined simultaneously in evaluating the oxidative stress status of a subject [51]. Extending previous results, our data demonstrate an independent TOS/TAC variation in patients and healthy individuals. The wide scattering of the oxidant-antioxidant balance, shown in all three groups of this study, supports the hypothesis of non-linearly dependent TOS/TAC changes. Phenotypic characteristics, such as the generated chronic low-grade inflammatory state by obesity, metabolic syndrome, smoking, and diabetes, or epigenetic changes, may unpredictably skew the TOS/TAC ratio [52]. It has previously been suggested that increased antioxidant enzyme activity in overweight children may be an effort to balance increased oxygen-free radical production [53,54].

*Innate Immunity—oxidative stress interplay:* There is currently enough evidence to support the critical interplay among various inflammatory and innate immunity mediators in promoting an oxidative stress microenvironment, and in regulating cell death [55,56]. It has previously been shown that septic patients with pronounced oxidative damage had increased Hsp72 serum levels, associated with an acute hormonal response by cortisol, prolactin, the human glucocorticoid receptor, and mortality [14,57,58]. It has also been demonstrated that the Hsp72 serum protein and mRNA expression in lymphocytes and monocytes were correlated to serum interleukins in sepsis [59] and that glutamine could repress intracellular Hsp72 after lipopolysaccharide (LPS) exposure [60]. In this study, cytokines TNF, IL-6, IL-10, IFN-γ, IL-27, alarmins Hsp72 and Hsp90, and markers associated with inflammation (CRP, procalcitonin, lactate) and stress (glucose) exhibited early-onset induction, which was strongly correlated with an increased TOS/TAC ratio in sepsis. Regarding lactate, it is now thought to be produced by cells in stress and probably offers some form of protection, by promoting resistance to oxidative stress [61]. Even though lactate has long been viewed as a metabolic waste product, it is now considered an important signaling molecule and an inter-cellular redox carrier, helping to maintain tissue homeostasis and integrity [62]. Supporting our results, the strong antioxidant, anti-inflammatory, and anti-apoptotic protective efficacy of protocatechuic acid alleviated the LPS-induced septic lung injury in an experimental study in mice [63].

*Apoptotic/Anti-apoptotic and Oxidant/Antioxidant pathways*: The molecular mechanisms of the interaction between oxidative stress and inflammation, and their interplay with different types of programmed cell death, are unclear [21]. Current literature supports the hypothesis that oxidative stress enhances apoptotic pathways, through diverse signaling pathways activated by mitochondrial membrane permeabilization, ultimately leading to caspase-3 activation [64,65]. In this study, we showed that caspase-9 and caspase-3 are simultaneously induced in early-onset sepsis. Caspase-9 is required for mitochondrial morphological changes and TOS production, by cleaving and activating Bid into tBid. Caspase-3 is required for efficient execution of apoptosis and inhibition of TOS production [66]. Recent reports in sepsis associate mortality with protracted periods of immune paralysis, induced by immune cell alterations and uncontrolled or excessive apoptotic depletion of immune cells [67,68]. It has previously been shown that apoptosis and oxidative stress are interrelated events, closely linked to multiple cardiovascular, metabolic, autoimmune, or neurodegenerative disorders [69,70]. In this study, caspase-3 and caspase-9 were also upregulated in septic patients, compared to patients with non-infectious critical illness or healthy controls, while caspase-9 was positively correlated to glutathione. However, what is shown for the first time is that the expression of three anti-apoptotic survivin splice variants was upregulated in peripheral blood leukocytes, which, along with elevated serum survivin protein, was associated with repressed TAC and mortality in sepsis. This is in agreement with the findings of a recent study, showing that seemingly antagonistic pathways of both pro- and anti-apoptotic genes are simultaneously upregulated early in the course of sepsis [29]. New therapeutic agents, increasing ATP production and the activities of mitochondrial complexes I and III in LPS-stimulated cardiomyocytes, have recently been found to reduce the LPS-induced levels of reactive oxygen species by suppressing cleaved caspase 3 activity [71] or by increasing the activities of antioxidant enzymes, glutathione peroxidase, superoxide dismutase, and GSH [72]. Similar scientific research, focused on the apoptotic/anti-apoptotic and oxidant/antioxidant pathways in sepsis, might reveal promising therapeutic targets and encourage novel interventions in the future.

## 5. Limitations of the Study

Some limitations of our study should be mentioned. First, the sepsis sample size did not allow the execution of robust sub-analyses of specific infectious categories in this study to further clarify the interplay among oxidative, inflammatory, or apoptotic/anti-apoptotic pathways that are activated during the septic cascade. Second, the studied groups represent a real ICU population, with unmatched age of septic patients (older) and trauma patients (younger). Older surgical patients in ICUs are mainly represented by cancer patients who meet exclusion criteria. We analyzed the effects of age and sex covariates in the oxidative stress parameters by a propensity probability analysis, which showed no significant effect of these covariates in TOS/TAC. In addition, a bivariate correlation of TOS/TAC with age in the sepsis group alone did not produce any significant correlation. A similar study in oxidative stress parameters showed no differences regarding age (<60 versus >60 years, *p* > 0.05) in patients with cancer [73]. Finally, further experiments will be needed to elucidate more on the expression of these pathways in different cell types that are crucially affected in sepsis.

## 6. Conclusions

Sepsis is independently associated with an increased oxidant-antioxidant imbalance compared to non-septic critically ill patients. The oxidant-antioxidant imbalance is an early-onset sepsis discriminator associated with mortality and accompanied by an augmentation of caspases of the intrinsic mitochondrial apoptotic pathway and apoptosis effect. The TOS/TAC imbalance is affected by various inflammatory biomolecules, specific innate immune system alarmins, the anti-apoptotic protein survivin, and sepsis-related severity. The wide scattering of individual TOS/TAC balances shown in this study probably indicates that a personalized antioxidant therapeutic approach should be applied based on yet undefined phenotypes.

## Figures and Tables

**Table 1:** Patients’ demographics, clinical and routine laboratory characteristics.

Characteristic	Control (H) (*n* = 89)	Trauma/Surgery (I) (*n* =112)	Sepsis (S) (*n* = 145)	*p ^#^*
Age (years), mean ± SD	40.3 ± 14.4	40.8 ± 15.7	62.2 ± 15.6	0.001
Sex (Female/Male), (%)	39/50 (44/56)	23/89 (20/80)	60/85 (41/59)	0.478
ICU LOS (days), mean ± SD		21 ± 20.8	25 ± 21.9	0.636
Mortality ICU, *n* (%)		9 (8)	42 (30) ^†^	0.001
APACHE score, mean ± SD		15 ± 5.6	22.4 ± 8.1 ^†^	0.001
SOFA score, mean ± SD		8.3 ± 2.7	10 ± 3 ^†^	0.001
SAPS score, mean ± SD		48.8 ± 9.9	71.5 ± 13.3 ^†^	0.001
WBC × 10^3^ (cells/μL), median (IQR)		12.7 (9.5–16.7)	13.9 (8.6–20)	0.037
Lactate (mg/dL), median (IQR)		4.3 (2–13)	4.7 (1.9–23)	0.013
Glucose (mg/dL), median (IQR)		147 (129–189)	195 (134–267) ^†^	0.003
Albumin (mg/dL), median (IQR)		3.1 (2.7–3.5)	2.6 (2.3–2.9) ^†^	0.001
Urea (mg/dL), median (IQR)		28 (20–39)	83 (48.2–133) ^†^	0.001
Creatinine (mg/dL), median (IQR)		0.81 (0.7–1.1)	1.72 (1.1–2.9) ^†^	0.001
CRP (mg/dL) median (IQR)		9.9 (4.1–37.4)	25.6 (13–89) ^†^	0.001
Procalcitonin (ng/mL), median (IQR)		0.77 (0.44–1.82)	5 (1.14–28.8) ^†^	0.011

^#^ Differences among groups (ANOVA, Kruskal–Wallis, x^2^ test, as appropriate): Post hoc differences: ^†^ SIRS-sepsis. SD = standard deviation, IQR = Interquartile Range, ICU = Intensive Care Unit, LOS = Length of Stay, WBC = White Blood Cells, CRP = C-Reacting Protein.

**Table 2:** Characteristic biomolecules representing oxidative stress, innate immunity, and inflammatory and apoptotic/anti-apoptotic cascades.

Characteristic	Control (H) (*n* = 89)	Trauma/Surgery (I) (*n* =112)	Sepsis (S) (*n* = 145)	*p ^#^*
TOS/TAC, median (IQR)	0.82 (0.40–1.40) **	1.49 (0.81–2.20) *	8.90 (4.05–24.9) ^†^	0.001
TOS (μmol/L), median (IQR)	206 (99–340) **	315.6 (175–510) *	1222 (493–2022) ^†^	0.001
TAC (μmol/L), median (IQR)	218 (188–320) **	207 (172–288.5)	138.8 (63.5–190) ^†^	0.001
IL-6 (pg/mL), median (IQR)	3.5 (1.24–14.8) **	77 (19–157) *	86 (27–399) ^†^	0.001
IL-8 (pg/mL), median (IQR)	53.5 (25.5–154.7)	50.5 (28–96)	126.4 (56.4–233)	0.083
IL-10 (pg/mL), median (IQR)	3.7 (0.4–11.5) **	10.2 (0.01–25)	16.7 (5.2–68.7) ^†^	0.001
IL-27 (pg/mL), median (IQR)	0.4 (0.21–0.6) **	0.27 (0.2–0.7)	0.51 (0.25–0.9) ^†^	0.009
IL-17 (pg/mL), median (IQR)	0.77 (0.2–33)	0.75 (0.2–7.9)	2.4 (0.2–8.5)	0.711
Hsp72 (ng/mL), median (IQR)	0.2 (0.1–0.4) **	0.22 (0.12–0.4)	0.67 (0.2–1.6) **	0.001
Hsp90 (ng/mL), median (IQR)	43.8 (13.6–76.6) **	45.5 (26.2–106)	75.7 (38.2–183) **	0.001
IFN-γ (IU/mL), median (IQR)	5.3 (0.53–9.7)	4.6 (0.13–7.9)	8.9 (3.2–15.2) **	0.007
TNF-α (pg/mL), median (IQR)	4.36 (2.1–12.7)	7.8 (3.6–259) **	25.4 (6.1–332.5)	0.016
Zinc (μg/dl), median (IQR)	80 (72–93) **	49.5 (35–56.2)	49.5 (37–60.7) **	0.001
Glutamine (μmol/L), mean ± SE	460 ± 238	478 ± 153	492 ± 162	0.602
Glutathione (μmol/L), median (IQR)	750 (550–800) **	650 (550–900)	825 (650–1050) **	0.029
Survivin protein (pg/mL), median (IQR)	108 (28.6–180) **	140 (99.9–228)	179 (125–420) **	0.001
Caspase-3 (ng/mL), median (IQR)	17.3 (9.9–44.4)	11.3 (2.6–37.6)	49.8 (9.3–90) **	0.021
Caspase-9 (ng/mL), median (IQR)	91.8 (80.7–105) **	92.7 (86–119)	112 (95.3–146)	0.014
Survivin-WT isoform (copies/μL), median (IQR)	0.005 (0.001–0.015) **	0.006 (0.001–0.019)	0.046 (0.005–0.2) **	0.001
Survivin-2B isoform (copies/μL), median (IQR)	5.7 (2.4–11.3)	10.9 (6.7–18.2)	20.6 9.2–35.4) **	0.001
Survivin-ΔΕx3 isoform (copies/μL), median (IQR)	0.005 (0.003–0.016)	0.011 (0.003–0.034)	0.1 (0.012–0.36) **	0.001
Survivin-3B isoform (copies/μL), median (IQR)	0.1 (0.01–0.25)	0.26 (0.03–0.57) **	0.11 (0.02–0.27) **	0.029

^#^ Differences among groups (ANOVA, Kruskal–Wallis, x^2^ test, as appropriate): Post hoc differences: * control-SIRS, ** control-sepsis, ^†^ SIRS-sepsis. TOS = Total Oxidative Stress, TAC = Total antioxidant capacity, IQR = Interquartile Range, IL = Interleukin, IFN-γ = interferon gamma, TNF = tumor necrosis factor.

**Table 3:** Bivariate correlations for TOS and TAC in the septic group (S).

	TOS/TAC	TOS	TAC
		Spearman’s r (*p*-Value)	-
Age	−0.16 (0.116)	0.44 (0.671)	−0.19 (0.083)
SOFA score	0.22 (0.001)	0.22 (0.003)	−0.16 (0.027)
APACHE score	0.216 (0.001)	0.22 (0.006)	−0.17 (0.029)
SAPS score	0.366 (0.001)	0.37 (0.001)	−0.37 (0.001)
CRP	0.215 (0.008)	0.17 (0.03)	−0.23 (0.003)
Procalcitonin	0.56 (0.002)	0.5 (0.007)	−0.5 (0.008)
IL-6	0.38 (0.001)	0.35 (0.001)	−0.28 (0.001)
IL-10	0.28 (0.001)	0.224 (0.001)	−0.18 (0.007)
IL-27	0.21 (0.009)	0.226 (0.005)	−0.08 (0.3)
IFN-γ	0.31 (0.001)	0.34 (0.001)	−0.08 (0.35)
Hsp72	0.3 (0.001)	0.25 (0.001)	−0.32 (0.001)
Hsp90	0.2 (0.001)	0.18 (0.004)	−0.13 (0.042)
Survivin protein	0.19 (0.009)	0.16 (0.026)	−0.17 (0.019)
Caspase-3	0.24 (0.088)	0.17 (0.35)	−0.2 (0.13)
Caspase-9	0.16 (0.232)	0.13 (0.35)	−0.1 (0.4)
Survivin-WT	0.32 (0.001)	0.3 (0.002)	−0.14 (0.14)
Survivin-2B	0.34 (0.001)	0.34 (0.001)	−0.19 (0.018)
Survivin-ΔΕx3	0.4 (0.001)	0.4 (0.001)	−0.2 (0.012)
Survivin-3B	−0.10 (0.28)	−0.12 (0.21)	−0.27 (0.76)
Zinc (Zn)	−0.39 (0.001)	−0.37 (0.001)	0.22 (0.03)

Bivariate correlations describing possible associations of TOS/TAC ratio with demographic, clinical, and laboratory parameters of the study. Statistical significance was defined according to the 95% confidence level. TOS = total oxidate status, TAC = total antioxidant capacity, Hsp = heat shock protein, IL = interleukins, IFN-γ = interferon gamma, CRP = C-reactive protein.

**Table 4:** Area Under the Curve for discriminating sepsis among critically ill patients.

				Asymptotic 95% Confidence Interval	
Test Result Variable(s)	Area	Std. Error	Asymptotic Sig.	Lower Bound	Upper Bound
TOS/TAC	0.958	0.015	<0.000	0.928	0.988
Hsp90	0.666	0.048	0.001	0.572	0.761
Hsp72	0.712	0.045	<0.000	0.625	0.800
TOS	0.901	0.026	<0.000	0.851	0.952
SOFA score	0.711	0.045	<0.000	0.623	0.799
Survivin protein	0.618	0.049	0.024	0.521	0.715
TAC	−0.852	0.033	<0.000	0.084	0.213

**Table 5:** Oxidative, inflammatory, and apoptotic/anti-apoptotic levels related to mortality.

Laboratory Assay	Survival	Mortality	*p ^#^*
TOS/TAC ratio, median (IQR)	1.52 (0.7–3)	7.5 (3.2–26)	0.001
TOS, median (IQR)	318 (172.5–568)	1243 (505–2104)	0.001
TAC, median (IQR)	197 (160–257)	142 (72–258)	0.001
IL-6 (pg/mL), median (IQR)	32 (3.7–130)	64 (26–310)	0.002
IL-8 (pg/mL), median (IQR)	60.6 (30–168)	77.8 (51.4–126)	0.785
IL-10 (pg/mL), median (IQR)	9 (1.2–20)	13.9 (1.5–55)	0.148
IL-27 (pg/mL), median (IQR)	0.4 (0.2–0.7)	0.6 (0.26–0.95)	0.015
Hsp72 (ng/mL), median (IQR)	0.27 (0.14–0.70)	0.58 (0.24–1.5)	0.001
Hsp90 (ng/mL), median (IQR)	54.8 (25.4–117)	62.4 (35–146)	0.105
IFN-γ (IU/mL), median (IQR)	5.1 (0.6–10.3)	6.69 (3.15–13.3)	0.196
TNF (pg/mL), median (IQR)	7.85 (3. 6–67.7)	4.62 (4.4–4.8)	0.543
Zinc (μg/dL), median (IQR)	56 (41–76)	55.5 (43–66)	0.409
Survivin-WT isoform (copies/μL), median (IQR)	0.007 (0.002–0.03)	0.022 (0.004–0.27)	0.019
Survivin-2B isoform (copies/μL), median (IQR)	0.13 (0.01–0.3)	0.16 (0.04–0.35)	0.015
Survivin protein (pg/mL), median (IQR)	136.7 (84–240)	179 (130–424)	0.002
Caspase-3 (ng/mL), median (IQR)	21.1 (4.5–55.4)	15.2 (5.1–92.6)	0.674
Caspase-9 (ng/mL), median (IQR)	102 (87.5–136.5)	99 (90.3–123.4)	0.784

# Differences among groups (Mann–Whitney U-test): TOS = Total Oxidative Stress, TAC = Total antioxidant capacity, IQR = Interquartile Range, TNF = tumor necrosis factor, Hsp = heat shock protein, IL = interleukins, IFN-γ = interferon gamma, TNF = tumor necrosis factor.

**Table 6:** Area Under the Curve for predicting mortality in septic patients.

				Asymptotic 95% Confidence Interval	
Test Result Variable (s)	Area	Std. Error	Asymptotic Sig.	Lower Bound	Upper Bound
TOS/TAC	0.801	0.046	<0.001	0.710	0.892
TOS	0.732	0.058	0.002	0.619	0.846
Survivin protein	0.720	0.069	0.003	0.585	0.855
Survivin-WT	0.705	0.071	0.006	0.565	0.845
Survivin-2B	0.735	0.055	0.002	0.627	0.844
Hsp90	0.687	0.060	0.013	0.569	0.805
IL-6	0.720	0.063	0.003	0.597	0.843
TAC	−0.758	0.057	0.001	0.131	0.354

## Data Availability

The data presented in the study are all contained within this article.

## Supplementary Materials

The following supporting information can be downloaded at: https://www.mdpi.com/article/10.3390/antiox11020231/s1, Table S1: Propensity probability logistic regression model adjusted for predicted probabilities of covariates age and sex.
