# Supplementary material for: Oxidant/Antioxidant Status Is Impaired in Sepsis and Is Related to Anti-Apoptotic, Inflammatory, and Innate Immunity Alterations
Source: Antioxidants (Basel). 2022 Jan 25;11(2):231. doi: 10.3390/antiox11020231 (PMC8868413; doi:10.3390/antiox11020231)
Supplement: Supplementary file 1 [file antioxidants-11-00231-s001.zip › antioxidants-1430925-supplementary.pdf]

antioxidants

MDPI

Supplemental Table S1. Propensity probability logistic regression model adjusted for predicted probabilities of covariates age and sex. Dependent variable: TOS/TAC defined by the "optimal" cut off point TOS/TAC value; independent variables: predicted probability of age and sex, diagnostic categories (sepsis, non-septic ICU patients, and healthy controls), the severity of illness (SOFA score) and alarmin Hsp72.

Variables in the Equation

		B	S.E.	Wald	df	Sig.	Exp(B)	95% C.I.for EXP(B)	
								Lower	Upper
Step 1	Diagnosis_code	3.236	.566	32.637	1	<.001	25.429	8.379	77.172
	HSP72	.179	.156	1.321	1	.250	1.196	.881	1.623
	Predicted probability	-.809	.930	.756	1	.385	.446	.072	2.756
	SOFA score	-.038	.071	.287	1	.592	.963	.838	1.106
	Constant	-7.633	1.286	35.211	1	<.001	.000		
